# Public Health Administration in Australia

**Published:** 1920-04-03

**Authors:** 


### PUBLIC HEALTH ADMINISTRATION IN AUSTRALIA.

BY OUR SPECIAL CORRESPONDENT.

The new Health Bill now before the Victorian Legislative Assembly promises to bring about some radical changes in public health administration. For instance, the amended definition of a boarding-house is any house, tent, or other structure in which more than five persons, exclusive of the proprietor's family, are lodged or boarded; while the definition of a "home" has been extended to include "workroom." A clause has been inserted, on the motion of the Premier (Mr. Lawson), that the Governor in Council may make regulations from time to time for the destruction of rats and mice. A somewhat drastic clause provides that the Minister of Health may arrange with the managing authorities of hospitals or with medical practitioners or dentists for the treatment of physical defects discovered in school children by medical inspection. The Premier, who favoured this clause, admitted it would mean "almost a revolution." One clause empowers the Minister to recover from municipalities the cost of dealing with infectious diseases, when the municipality failed to deal adequately with the disease.

An attempt is to be made in the new Health Bill to overcome the difficulty between the Victorian Branch of the B.M.A. and the friendly societies in regard to lodge patients, which came about two or three years ago, and, notwithstanding a Court judgment in favour of the former's claims, still continues. This dispute has given rise to much ill-feeling among the parties concerned. That there were faults on both sides is the general opinion of outsiders. The clause in question gives power to the Government or municipalities "to appoint medical officers for the purposes of the section, the remuneration to be such as is agreed upon." The duties of these medical officers would be to give medical attendance to persons entitled to receive it, pursuant to the rules of any registered friendly society which entered into an agreement with the Minister or the municipality. The medical men were, further, to perform such other duties as might be agreed upon or prescribed. Power was given to make regulations for the duties of officers, the terms and conditions of contracts, and the rates of payments to be made by the societies for the services.

Commenting on this action of the State Ministry in meddling in the dispute between friendly societies and medical men, the *Melbourne Argus* of December 5 says: "The Ministry has no more right to interfere between a citizen and his doctor than between a citizen and his clergyman, and it is a monstrous misuse of power to interfere in such a way as to give some citizens special benefits at the expense of other citizens. Apart from the practical difficulties—for the proposal will involve the appointment of hundreds of medical officers all over the State—the violation of the principle of equal treatment for all people cannot be too strongly condemned." One anti-B.M.A. member of the House submitted a black list issued by the Association, which had been distributed among the medical profession and nursing homes. This list contained the names of seventy-six doctors because they had entered into agreement with friendly societies contrary to the direction of the B.M.A. The doctors named were all qualified men practising in Victoria, and included every homœopathic practitioner. Needless to say, the friendly societies are pleased with the turn of events, which is more than can be said for members of the Victorian Branch of the B.M.A.

